# Does treatment with sodium-glucose co-transporter-2 inhibitors have an effect on sleep quality, quality of life, and anxiety levels in people with Type 2 diabetes mellitus?

**DOI:** 10.3906/sag-2008-37

**Published:** 2021-04-30

**Authors:** Serdar ŞAHİN, Özlem HALİLOĞLU, Özge POLAT KORKMAZ, Emre DURCAN, Hümeyra REKALI ŞAHİN, Volkan Demirhan YUMUK, Taner DAMCI, Hasan İLKOVA, Zeynep OŞAR SİVA

**Affiliations:** 1 1Department of Internal Medicine, Faculty of Medicine, İstanbul University-Cerrahpasa, İstanbul, Turkey; 2 Department of Internal Medicine, Taksim Training and Research Hospital, İstanbul Turkey

**Keywords:** Anxiety, sleep quality, quality of life, sodium-glucose co-transporter 2 inhibitor

## Abstract

**Background/aim:**

To evaluate the impact of treatment with sodium-glucose co-transporter-2 (SGLT2) Inhibitors on quality of life (QoL), sleep quality (SQ), and anxiety levels in patients with Type 2 diabetes mellitus (T2DM).

**Materials and methods:**

Ninety-seven patients with type 2 diabetes admitted to tertiary care hospital diabetes clinic were included. Fifty patients were randomized to receive SGLT2 inhibitors in addition to baseline treatment (Group A), 47 subjects continued with their baseline treatment or were added other medications as needed (Group B). Thirty healthy controls (HC) were recruited (Group C). All groups were subjected to the Turkish version of Short Form-36 (SF-36), Pittsburgh Sleep Quality (PSQ), and Beck Anxiety Inventory (BAI) scales both at baseline and final visit.

**Results:**

Physical function, emotional role limitation, vitality, mental health, pain, general health perception scores of SF-36 were significantly improved in Group A, at the end of the follow-up period. There was no significant change in terms of PSQ, BAI scores, and hypoglycaemia documented in all groups. The intervention-related change in HbA1c level, body weight, and body mass index were significantly higher in Group A.

**Conclusion:**

The QoL was improved in people with diabetes who were taking SGLT2 inhibitors. This may be explained by weight loss observed in participants.

## 1. Introduction

SGLT2 inhibitors are glucose lowering drugs with proven effect both on fasting and postprandial blood glucose levels [1–3]. They decrease plasma glucose levels by inhibiting tubular glucose reabsorption and increasing urinary glucose excretion [3]. Recent evidence of large randomized controlled trials supports the role of SGLT2 inhibitors as a preferred second and possibly first line drug in diabetic subjects especially with established atherosclerotic cardiovascular disease (ASCVD) and/or renal complications [4]. The SGLT2 inhibitor agents empagliflozin and dapagliflozin both have been shown to reduce hospitalization rate for heart failure and improve renal outcome in subjects with T2DM, who had or were at a high risk for ASCVD [5,6]. The SGLT2 inhibitors are generally well tolerated drugs [7]. 

The assessment of QoL in patients with T2DM is regarded as an important part of diabetes care [8]. There is clear evidence that patients with diabetes have a lower quality of life (QoL) compared to nondiabetic subjects [9–11]. Therefore, QoL indexes are important tools used in the evaluation of efficacy and tolerability of different treatment modalities in diabetes [12]. One of the various scales used in this regard is SF-36, the health-related QoL index [8] that may provide important information in clinical decision-making [12]. 

In recent studies a positive effect of SGLT2 inhibitors on QoL has been documented, but there is insufficient data about the treatment associated effects on SQ or anxiety [13,14]. In this study, we aimed to evaluate the effects of the treatment with SGLT2 inhibitors on QoL, sleep quality (SQ), and anxiety levels of patients with T2DM. The primary endpoints were changes in physical and mental components of QoL, SQ, and anxiety score from baseline to the end of the treatment period. 

## 2. Material and methods

### 2.1. Study population

Ninety-seven patients with T2DM presented consecutively to the diabetes clinic between May 2019 and January 2020 were included. In this study, fifty patients were randomized to receive empagliflozin 10 mg or dapagliflozin 10 mg OD in addition to their baseline treatment (Group A), and 47 patients continued their baseline treatment or were added other medications (except SGLT-2 inhibitors) as needed (Group B). Thirty age and sex matched healthy controls (HC) were recruited from patients’ relatives (Group C). All patients were given diet and lifestyle counseling for type 2 diabetes throughout the study. Eligible patients were 18 years of age, not on SGLT2 inhibitors, had HbA1c level >7% and eGFR > 60mL/min/1.73m2. Patients were excluded if they were pregnant, had Type 1 diabetes mellitus, hypothyroidism, cancer, depression, chronic obstructive pulmonary disease, sleep disturbances, benign prostatic hyperplasia, and any symptom suggestive for genitourinary disease. 

### 2.2. Study procedure

All groups were evaluated using the Turkish version of SF-36, PSQ, and BAI scale initially and at the third month of the treatment period. A self-measured blood glucose (SMBG) reading ≤70 mg/dL or occurrence any of the hypoglycaemia related symptoms was defined as hypoglycaemia [15]. Weekly frequency of hypoglycaemia, daily and nocturnal urination frequencies and symptoms related to urinary tract infection (UTI), genital infection (GI) or urinary incontinence occurring in the last month previous to randomization and in the treatment period were evaluated by the same diabetes nurse. Body weight, body mass index (BMI), and HbA1c levels were also measured at baseline and at the third month of follow-up. We used high performance liquid chromatography method for the determination of HbA1c. 

SF-36, Pittsburgh Sleep Quality (PSQ) and Beck Anxiety Inventory (BAI) scores, body mass index (BMI), frequencies of daily and nocturnal urination, urinary incontinence, and episodes of genitourinary tract infections at baseline and at the end of the treatment period were compared within all groups. HbA1c levels and hypoglycaemia documented also were compared between the intervention groups.

### 2.3. Questionnaires

SF-36 is a self-assessment scale that consists of 36 items and eight sub-dimensions. The physical component of the SF-36 evaluates physical function, physical role limitation, pain, and general health perception, whereas its mental component assesses emotional role limitation, vitality, mental health, and social functionality [16]. In the SF-36, the lower score reflected a poorer QoL. The Turkish version of the SF-36 has been validated by Koçyiğit et al. [17]. The PSQ evaluates the nocturnal SQ and daytime sleepiness. The Turkish version of the PSQ has been validated by Ağargün et al. [18]. BAI is a scale consists of 21 questions evaluating the frequency and severity of anxiety symptoms. The Turkish version of BAI has been validated by Ulusoy et al. [19]. 

### 2.4. Statistical analysis

A database was created with the data for all variables, and the data were analysed with the SPSS v.22.0 software (IBM Corp., New York, Armonk, NY, USA). The confidence level was set at 95%. The distribution of variables was measured using the Kolmogorov–Smirnov test. Continuous variables were expressed as means ± standard deviation in normal distribution, as medians [interquartile range (IQR)] in nonnormal distribution. In the comparisons among groups, for the analysis of quantitative independent data, we performed the ANOVA test in normal distribution, and in posthoc, we performed the Tukey test. In nonnormal distribution, we used the Kruskal–Wallis and Mann–Whitney U test for the analysis of quantitative independent data. Bonferroni correction was then performed to account errors due to the multiple comparisons among groups. In nonnormal distribution and quantitative dependent data, we used the Wilcoxon test, and in normal distribution and quantitative dependent data, we used paired sample t-test. Fisher’s exact Chi-square (χ2) test was used for the analysis of qualitative independent data. Spearman’s rank-order test was used to calculate the correlation coefficients between continuous variables.

This study was approved by the local ethics committee. The study adhered to the tenets of Helsinki. Written informed consent was obtained from all participants before they enrolled.

## 3. Results

We included 97 patients with T2DM and thirty HCs in the study. The demographic data and general characteristics of the subjects are presented in Table 1. There was a significant decrease in HbA1c, body weight, and BMI values in Group A (P = 0.001, P < 0.001, P < 0.001, respectively), in body weight in Group B (P = 0.010) at the end of the follow-up period (Table 2). In the last month previous to randomization and during the study, we did not find any significant difference in weekly frequency of hypoglycaemia in intervention groups (P = 0.678, P = 0.495 respectively).

**Table 1:** Baseline characteristics of the patients and healthy controls.

	Group A	Group B	Group C	P§
Sex (F/M) (n; %)	23/27	28/19	20/10	0.161
Age (years) (Mean±SD)	52.9 8.72	55.9.52	52.1 5.11	0.185
HbA1c (%) [Median (IQR)]	8.3 (7.6–9.6)	8.1 (7.0–9.0)	-	0.224
Duration of DM (years) [median (IQR)]	10.0 (7.7–15.2)	10.0 (6.0–15.0)	-	0.147
Complications (n; %) (n; %)				
Nephropathy	15 (30)	8 (17)	-	0.133
Retinopathy	7 (14)	6 (12)	-	0.858
Neuropathy	17 (34)	8 (17)	-	0.056
Ischemic heart disease	5 (10)	9 (19)	-	0.200
Hypertension	31 (62)	28 (59)	-	0.807
Hyperlipidemia	30 (60)	26 (55)	-	0.641
Peripheral vascular disease	6 (12)	1 (2)	-	0.113
Cerebrovascular disease	0 (0)	2 (4)	-	0.141
Antidiabetic drugs (n; %) (n; %)				
Sulphonylurea	12 (24)	6 (12)	-	0.155
DPP-4 inhibitors	37 (74)	25 (53)	-	0.033*
Metformin	40 (80)	43 (91)	-	0.108
Pioglitazone	2 (4)	1 (2)	-	0.594
Insulin	21 (42)	16 (34)	-	0.420

DM: Diabetes mellitus, DPP-4: Dipeptidyl peptidase-4 inhibitors, F: Female, M: Male§In the comparisons among groups A-B-C for the analysis of quantitative independent data, we used the ANOVA test in normal distribution and used the Tukey test in post-hoc. In the comparisons among groups A-B, for the analysis of quantitative independent data (HbA1c, duration of DM), we used the Mann–Whitney U in nonnormal distribution. Fisher’s exact Chi-square test was used for the analysis of qualitative independent data among groups. Continuous variables were expressed as mean ± standard deviation in normal distribution, as medians [interquartile range (IQR)] in nonnormal distribution.

**Table 2:** The comparison of SF-36, PSQ, BAI scores and BMI, body weight values documented at baseline and end of the study period within groups.

	Baseline	3rd month	P¶
Group A			
PSQ [median (IQR)]	4.0 (3.0–8.0)	4.5 (3.0–8.2)	1.000
BAI [median (IQR)]	5.5 (3.0–14.2)	5.0 (3.0–8.2)	0.245
Physical function [median (IQR)]	77.5 (55.0–90.0)	80.0 (65.0–96.2)	0.006*
Physical role limitation [median (IQR)]	100.0 (43.7–100.0)	100.0 (50.0–100.0)	0.118
Pain [median (IQR)]	88.7 (57.5–100.0)	90.0 (67.5–100.0)	<0.001*
General health perception [median (IQR)]	55.0 (33.7–70.0)	60.0 (43.7–76.2)	<0.001*
Emotional role limitation [median (IQR)]	83.3 (33.3–100.0)	100.0 (66.6–100.0)	0.040*
Vitality [median (IQR)]	50.0 (33.7–66.2)	62.5 (40.0–75.0)	<0.001*
Mental health [median (IQR)]	68.0 (48.0–80.0)	72.0 (55.0–84.0)	0.046*
Social functionality [median (IQR)]	87.5 (66.8–100.0)	87.5 (75.6–100.0)	0.184
BMI [median (IQR)]	32.0 (29.0–35.0)	31.0 (28.5–34.0)	<0.001*
Body weight [median (IQR)]	90.5 (80.7–100.0)	89.5 (79.7–96.2)	<0.001*
Group B			
PSQ [median (IQR)]	4.0 (2.0–7.0)	4.0 (2.0–7.0)	0.429
BAI [median (IQR)]	7.0 (2.0–13.0)	4.0 (2.0–7.0)	0.191
Physical function [median (IQR)]	80.0 (50.0–100.0)	75.0 (50.0–90.0)	0.063
Physical role limitation [median (IQR)]	100.0 (0.0–100.0)	100.0 (0.0–100.0)	1.000
Pain [median (IQR)]	87.5 (50.0–100.0)	87.5 (50.0–100.0)	0.775
General health perception (mean±SD)	64.418.0	65.117.9	0.135
Emotional role limitation [median (IQR)]	66.6 (33.3–100.0)	66.6 (33.3–100.0)	0.569
Vitality [median (IQR)]	65.0 (50.0–75.0)	60.0 (50.0–75.0)	0.569
Mental health (Mean±SD)	72.914.8	71.815.0	0.036*
Social functionality [median (IQR)]	90.0 (77.5–100.0)	87.5 (77.5–100.0)	0.569
BMI (Mean±SD)	30.694.8	30.624.7	0.134
Body weight (Mean±SD)	84.513.4	84.313.4	0.010*
Group C			
PSQ (Mean±SD)	5.23.0	5.12.8	0.184
BAI [median (IQR)]	7.0 (2.7–11.0)	7.0 (4.5–10.2)	0.712
Physical function [median (IQR)]	100.0 (78.7–100.0)	100.0 (78.7–100.0)	1.000
Physical role limitation [median (IQR)]	100.0 (100.0–100.0)	100.0 (100.0–100.0)	0.326
Pain [median (IQR)]	90.0 (67.5–100.0)	90.0 (67.5–100.0)	1.000
General health perception [median (IQR)]	75.0 (60.0–85.0)	75.0 (60.0–85.0)	0.326
Emotional role limitation [median (IQR)]	66.0 (66.0–100.0)	66.0 (66.0–100.0)	0.326
Vitality (mean±SD)	59.320.5	59.520.9	0.769
Mental health [median (IQR)]	74.0 (67.0–84.0)	76.0 (67.0–85.0)	0.246
Social functionality [median (IQR)]	100.0 (87.5–100.0)	100.0 (87.5–100.0)	0.214
BMI (mean±SD)	25.73.1	25.83.1	0.171
Body weight (Mean±SD)	70.911.0	71.111.0	0.134

BAI: Beck Anxiety Inventory, BMI: Body Mass Index, PSQ: Pittsburgh Sleep Quality, SF-36: Short Form-36¶Between baseline and 3rd month within each group, for comparisons of quantitative dependent data, we used the Wilcoxon test in nonnormal distribution and used paired sample t-test in normal distribution. Continuous variables were expressed as mean ± standard deviation in normal distribution, as medians [interquartile range (IQR)] in nonnormal distribution.*P < 0.05 was considered statistically significance.

When we evaluated the mental components of QoL, we found a significant increase in the emotional role limitation, vitality, and mental health scores in Group A (P = 0.040, P < 0.001, P = 0.046 respectively) at the end of the 3 months. In contrast, there was no significant increase in terms of mental components of SF-36 in the other groups (Table 2). The intervention-related changes in mental component, such as vitality was significantly higher in Group A compared to other groups (P = 0.004 vs. Group B, P = 0.004 vs. Group C). In addition, scores of mental health was higher in Group A compared to Group B (P = 0.001).

When we evaluated the physical components of QoL, in Group A, there was a significant increase in physical function, general health perception, pain scores at the end of the 3 months (P = 0.006, P < 0.001, and P < 0.001, respectively). In contrast, there was no significant increase in terms of physical components of SF-36 in the other groups (Table 2). The intervention-related changes in physical component, such as general health perception (P < 0.001 vs. Group B, P < 0.001 vs. Group C), physical function (P = 0.001 vs. Group B), and pain (P = 0.006 vs. Group B, P = 0.010 vs. Group C) were significantly higher in Group A compared to other groups. The comparison of the changes in the HbA1c values, BMI, SF-36-, PSQ-, and BAI-scores are presented in Table 3.

**Table 3:** The comparison of changes in the HbA1c and BMI, SF-36-, PSQ-, BAI scores among groups.

	Group A	Group B	Group C	P†
	[Median (IQR)]	[Median (IQR)]	[Median (IQR)]	
PSQ	0.0 (0.0–0.0)	0.0 (between –1.0 and 1.0 )	0.0 (0.0–0.0)	0.832
BAI	0.0(between –1.0 and 0.0)	0.0 (between–2.0 and 0.0)	0.0 (0.0–0.0)	0.285
SF-36				
Physical function	0.0 (0.0–11.2)a	0.0 (0.0–0.0)	0.0 (0.0–0.0)	0.002*
Physical role limitation	0.0 (0.0–0.0)	0.0 (0.0–0.0)	0.0 (0.0–0.0)	0.056
Pain	0.0 (0.0–10.0)b,c	0.0 (0.0–0.0)	0.0 (0.0–0.0)	0.002*
General health perception	5.0 (0.1–10.0)d,e	0.0 (0.0–0.0)	0.0(0.0–0.0)	<0.001*
Emotional role limitation f	0.0 (0.0–0.0)	0.0 (0.0–0.0)	0.0 (0.0–0.0)	0.032*
Vitality	0.0 (0.0–11.2)g,h	0.0 (0.0–0.0)	0.0 (0.0–0.0)	0.001*
Mental health	0.0 (0.0–9.0)i	0.0 (0.0–0.0)	0.0 (0.0–0.0)	0.002*
Social functionality	0.0 (0.0–0.0)	0.0 (0.0–0.0)	0.0 (0.0–0.0)	0.227
HbA1c	-0.4 (between–1.0 and –0.1)	0.1 (between–2.0 and 0.6)	-	< 0.001*
BMI	-0.3 (between–1.0 and 0.0)j,k	0.0 (0.0–0.0)l	0.0 (0.0–0.0)	<0.001*

BAI: Beck Anxiety Inventory, BMI: Body Mass Index, PSQ: Pittsburgh Sleep Quality, SF-36: Short Form-36†In the comparisons among groups, for the analysis of quantitative independent data, we used the ANOVA test in normal distribution and used the Tukey test in posthoc. In nonnormal distribution, we used the Kruskal–Wallis, and Mann–Whitney U test, Bonferroni correction was then performed to account errors due to the multiple comparisons among groups. In comparison among groups A-B, for the analysis of quantitative independent data (Hba1c), Mann–Whitney U was used in nonnormal distribution. Continuous variables were expressed as medians [interquartile range (IQR)] in nonnormal distribution.*P < 0.05 was considered statistically significance.Small letters were used for the pairwise comparisons among groups A-B-C.aP = 0.001 vs. Group B, bP = 0.006 vs. Group B, cP = 0.010 vs. Group C, dP < 0.001 vs. Group B, eP < 0.001 vs. Group C, f No statistical significance in pairwise comparisons, gP = 0.004 vs. Group B, hP = 0.004 vs. Group C, iP = 0.001 vs. Group B, jP = 0.016 vs. Group B, kP < 0.001 vs. Group C, l

In our study, poor SQ was found in 36 (37%) of 97 patients with T2DM and 11 (36%) of 30 HC. There were no statistically significant differences between the groups in terms of the PSQ, BAI scores, and rates of poor SQ. In Group A, there was a significant increase in GI (all documented fungal infections), episodes of urinary incontinence, nocturia, daily urination frequency, and UTI (P = 0.020, P = 0.008, P < 0.001, P < 0.001 and P = 0.035, respectively) compared to the other groups at the end of the study period. 

In Group A, the patients who had increased daily urination frequency and increased nocturia frequency weren’t shown any significant difference in terms of SF-36, PSQ, and BAI scores compared to those subjects without the urinary symptoms. Table 4 summarizes the correlation coefficients between the differences in HbA1c levels, BMI and SF-36-, PSQ- and BAI- scores in Group A. We did not observe any significant correlation between changes in HbA1c and in PSQ, in SF-36, and in BAI scores. However, there was a negative correlation between the treatment related changes in BMI and vitality, general health perception scores (P = 0.033; r = –0.304, P = 0.023, r = –0.323, respectively). Likewise, negative correlation was found between body weight and vitality, general health perception scores (P = 0.026; r = –0.314, P = 0.048, r = –0.282 respectively). 

**Table 4:** The correlation of changes in PSQ, BAI, and SF-36 scores with HbA1c levels, body weight, and BMI in Group A.

		r	#p
Pittsburgh sleep quality	HbA1c	0.147	0.331
	BMI	0.251	0.082
	Body weight	0.260	0.690
Beck anxiety	HbA1c	-0.044	0.772
	BMI	–0.013	0.928
	Body weight	0.027	0.854
SF–36 Physical function	HbA1c	0.000	0.999
	BMI	–0.236	0.102
	Body weight	–0.195	0.175
SF–36 Physical role limitation	HbA1c	–0.120	0.427
	BMI	–0.192	0.186
	Body weight	–0.197	0.170
SF–36 Pain	HbA1c	0.003	0.986
	BMI	–0.071	0.629
	Body weight	–0.054	0.709
SF–36 General health perception	HbA1c	–0.283	0.057
	BMI	–0.323	0.023*
	Body weight	–0.282	0.048*
SF–36 Emotional role limitation	HbA1c	–0.053	0.728
	BMI	–0.111	0.449
	Body weight	–0.112	0.438
SF–36 Vitality	HbA1c	–0.056	0.711
	BMI	–0.304	0.033*
	Body weight	–0.314	0.026*
SF–36 Mental health	HbA1c	–0.014	0.928
	BMI	–0.153	0.292
	Body weight	–0.169	0.241
SF–36 Social functionality	HbA1c	0.305	0.059
	BMI	0.057	0.695
	Body weight	–0.059	0.686

BAI: Beck Anxiety Inventory, BMI: Body Mass Index, PSQ: Pittsburgh Sleep Quality, SF-36: Short Form-36, *P < 0.05 was considered statistically significance. #Spearman’s rank-order test was used to calculate the correlation coefficients between continuous variables.

## 4. Discussion

In this prospective study, we found that the treatment with SGLT2 inhibitors is associated with a significant improvement in SF-36 measured QoL in patients with T2DM with poor glycemic control. In the SGLT2 inhibitor group (Group A), significant changes were documented both in mental and physical components of SF-36 after a three-month treatment period. Both HbA1c and BMI in this group decreased at the end of the treatment period. On the other hand, BMI correlated negatively with the treatment related changes in vitality and general health perception. The documented increases in urinary frequency, nocturia frequency, and genital infections did not have any effect on QoL, PSQ, and anxiety scores. 

Previous studies have shown that QoL is impaired especially in patients with T2DM with poor glycemic control [20,21], and the initiation of treatment with different antidiabetic drugs have a positive effect on QoL. Furthermore, blood glucose regulation significantly improved QoL in patients with type 2 diabetes [22,23]. In contrast, Hitoshi Ishii et al. reported that HbA1c was not correlated with the total diabetes therapy-related QoL score, but was significantly associated with the diabetes therapy-related satisfaction score [24]. We found similar results to previous studies in Group A. Although initiation of SGLT2 inhibitors significantly reduced HbA1c, the intervention related improvements in QoL scores did not show any correlation with the decreases in HbA1c. The lack of a significant correlation between improvements of QoL and change of HbA1c may be related with short follow up period, smaller sample size and the diversity of scales used. Even studies using SF-36 report variable effects of glycemic control on the QoL.

Previous studies showed that T2DM patients have poor SQ [25,26]. Moreover, some studies reported that SQ is worse in poorly controlled T2DM patients compared to well-controlled ones [27]. There are a few studies that reported a poor SQ prevalence between 55-71% in T2DM patients [26, 28, 29]. SQ was related to glycaemic control in T2DM patients [30]. In our study, the rates of patients with poor SQ were low when compared with the literature. Furthermore, although HbA1c decreased significantly in Group A, there was no significant change in SQ. The main reason that our results are not compatible with the literature may be the low number of patients with low SQ at the beginning of the study. In addition, we found no deterioration in SQ despite the increase in the nocturia frequency in the study group. 

*Collins et al.* detected that the prevalence of anxiety symptoms in patients with T2DM was 32% and established a relationship between poorly perceived glycaemic control and higher anxiety scores [31]. The other studies reported that the prevalence of anxiety was between 40%–58% in patients with T2DM [32–33]. In our study, the rate of patients with T2DM with anxiety symptoms was lower in Group A compared to the previous studies. Weight loss was a positive effect on QoL [34–36]. We found similar results in our study. Although previous studies showed that nocturia impairs SQ [37,38], it has been observed that SQ has not deteriorated in our study. The positive effects on QoL and weight in opposite the nocturia may have balanced the negative effect of nocturia on SQ.

In the literature, an association between hypoglycaemia and low QoL in T2DM was reported [39,40]. In our study, there was no significant change in hypoglycaemia frequency though the HbA1c levels decreased in Group A. We know that the risk of hypoglycaemic events during SGLT2 inhibitor therapy is generally low [41]. Moreover, a meta-analysis showed that hypoglycaemia was increased in canagliflozin treatment, whereas it was not increased in dapagliflozin or empagliflozin treatments [42]. 

Current studies have shown that SGLT2 inhibition increases the risk of GI, whereas their impact on the risk of UTI is unclear [43,44]. In our study, there was a significant increase in GI and UTI in Group A, but not in other groups. There was also a significant increase in UTI compared to the HCs, but no significant difference was observed when compared with Group B subjects. Weir MR et al. also reported increased frequency of osmotic diuresis-related events such as pollakiuria and polyuria after canagliflozin treatment [45]. On the other hand, L. H. Chen et al. showed that nocturia did not increase significantly in the dapagliflozin arm. Very few patients experienced nocturia with the evening dose but none with the morning dose [46]. In our study, the increase in nocturia may be due to the timing of SGLT 2 inhibitors.

The small number of patients, and the short follow-up period may be considered as the limitations to our study. On the other hand, the effect of SGLT2 inhibitor on anxiety, and SQ was examined for the first time in our study.

In conclusion, SGLT2 inhibitor-induced weight loss may be related to improvement in the QoL in people with T2DM. Despite the fact that it increases the nocturia frequency, daily urinary frequency, and GI SGLT2 inhibitors did not worsen the SQ or anxiety symptoms.

## Informed consent

Written informed consent was obtained from all participants before being enrolled.
